# Supplementary material for: Loss of Nnt Increases Expression of Oxidative Phosphorylation Complexes in C57BL/6J Hearts
Source: Int J Mol Sci. 2021 Jun 5;22(11):6101. doi: 10.3390/ijms22116101 (PMC8201288; doi:10.3390/ijms22116101)
Supplement: Supplementary file 1 [file ijms-22-06101-s001.zip › legend.pdf]

Figure S1. Gene expression of strain-dependent and Nnt-dependent genes in the adrenal. (A) Heatmap of 53 strain-dependent genes (significant DEGs in B6N vs. B6J and B6N vs. B6JBAC). (B) Heatmap of 7 Nnt-dependent genes (significant DEGs in B6N vs. B6J and B6JBAC vs. B6J. Rows show hierarchical clustering of DEGs, columns show samples. **Figure S2. Increased expression of electron transport chain genes in B6J and B6JBAC adrenals.** Heatmap of electron transport chain genes in adrenal glands from B6N, B6J and B6JBAC mice. Rows show hierarchical clustering of DEGs, columns show samples. Gene names omitted due to too many fields. White entries indicate no expression for that gene. **Figure S3. Gene expression of strain-dependent genes in the testes.** Heatmap of 201 strain-dependent genes (significant DEGs in B6N vs. B6J and B6N vs. B6JBAC). Rows show hierarchical clustering of DEGs, columns show samples. Gene names omitted due to too many fields. **Table S1.** Differential expression analysis of B6N and B6J hearts. *Output file of Deseq2 analysis of B6N and B6J hearts.* **Table S2.** Differential expression analysis of B6N and B6JBAC hearts. *Output file of Deseq2 analysis of B6N and B6JBAC hearts.* **Table S3.** Differential expression analysis of B6J and B6JBAC hearts. *Output file of Deseq2 analysis of B6J and B6JBAC hearts.* **Table S4.** Differential expression analysis of B6N and B6J adrenals. *Output file of Deseq2 analysis of B6N and B6J adrenals.* **Table S5.** Differential expression analysis of B6N and B6JBAC adrenals. *Output file of Deseq2 analysis of B6N and B6JBAC adrenals.* **Table S6.** Differential expression analysis of B6J and B6JBAC adrenals. *Output file of Deseq2 analysis of B6J and B6JBAC adrenals.* **Table S7.** Differential expression analysis of B6N and B6J testes. *Output file of Deseq2 analysis of B6N and B6J testes.* **Table S8.** Differential expression analysis of B6N and B6JBAC testes. *Output file of Deseq2 analysis of B6N and B6JBAC testes.* **Table S9.** Differential expression analysis of B6J and B6JBAC testes. *Output file of Deseq2 analysis of B6J and B6JBAC testes.*
