# Supplementary figures and images for: Loss of Nnt Increases Expression of Oxidative Phosphorylation Complexes in C57BL/6J Hearts
Source: Int J Mol Sci. 2021 Jun 5;22(11):6101. doi: 10.3390/ijms22116101 (PMC8201288; doi:10.3390/ijms22116101)

A

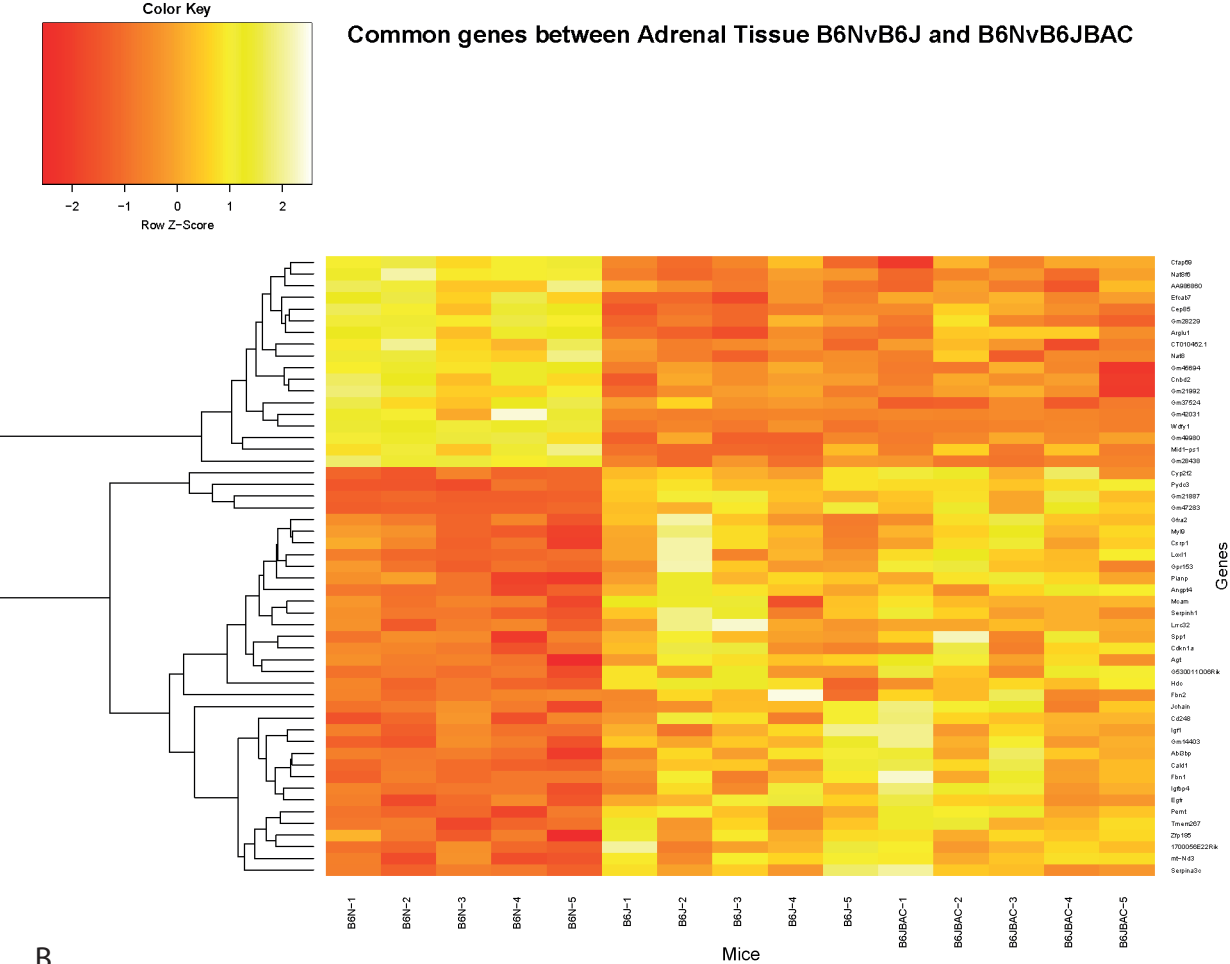

B

Supplement: Supplementary file 1 [file ijms-22-06101-s001.zip › Figure S1.pdf]

Electron Transport Genes in Adrenal Tissue

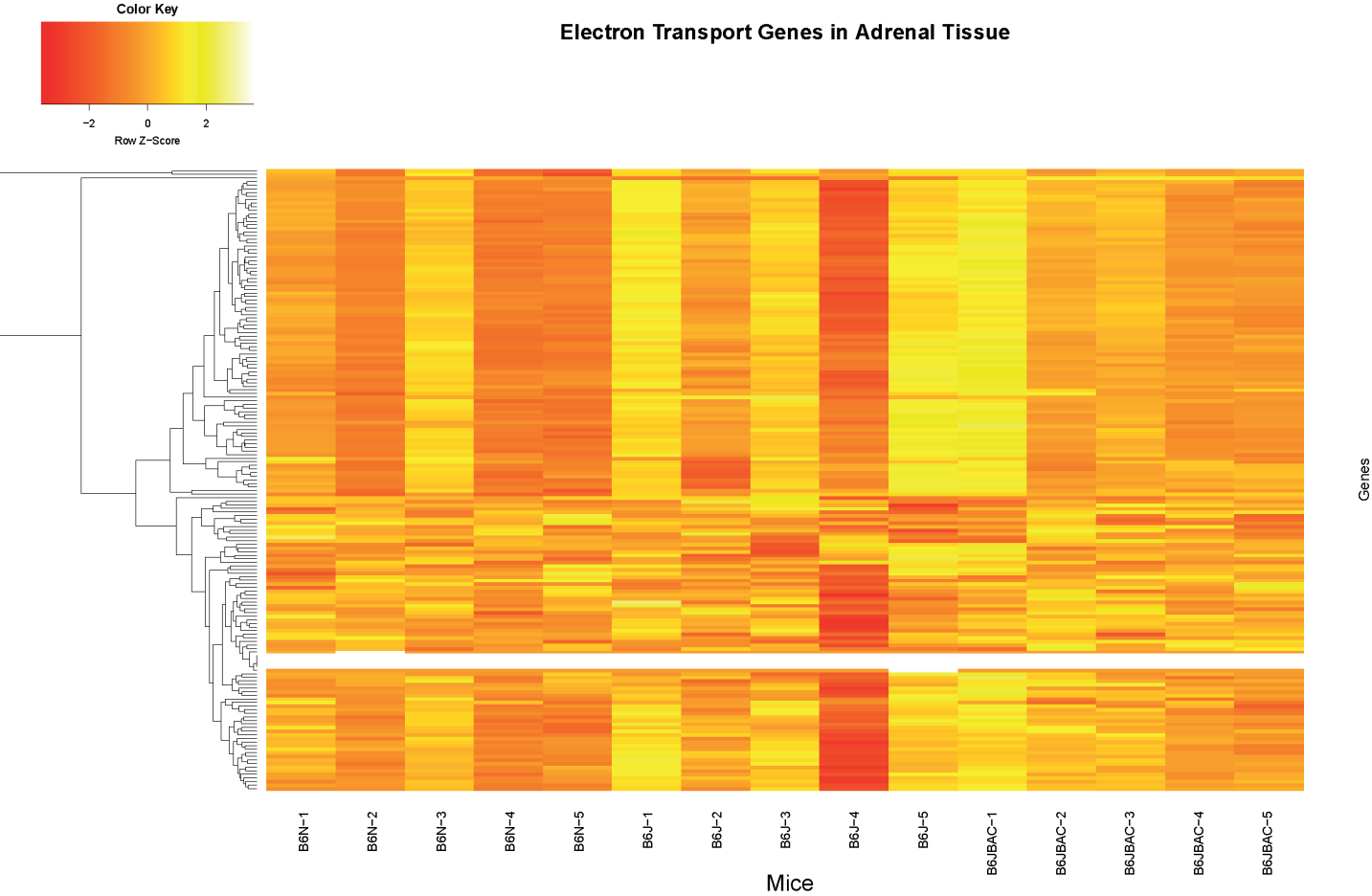

Supplement: Supplementary file 1 [file ijms-22-06101-s001.zip › Figure S2.pdf]

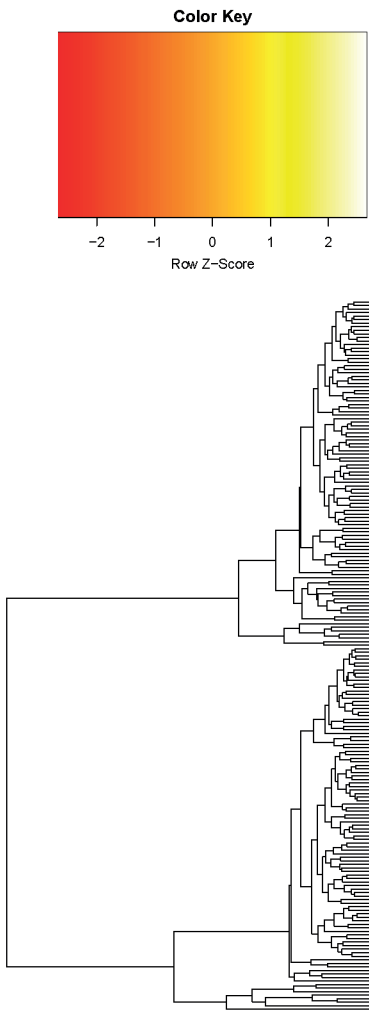

**Common genes between Testes Tissue B6NvB6J and B6NvB6JBAC**

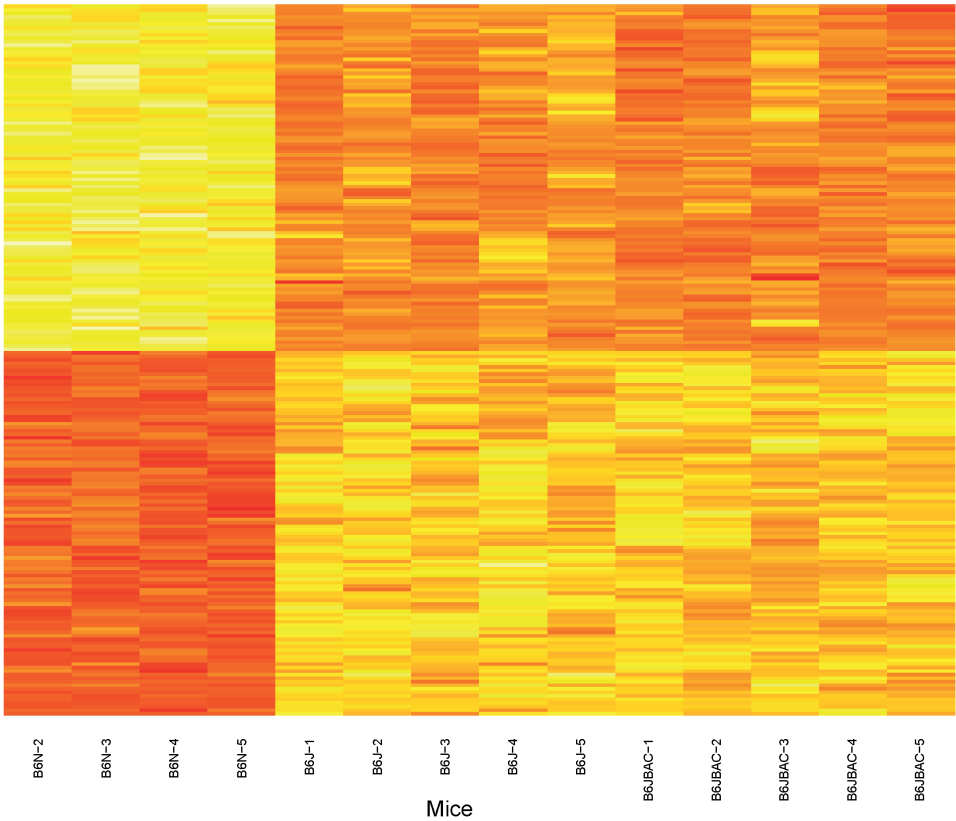

Supplement: Supplementary file 1 [file ijms-22-06101-s001.zip › Figure S3.pdf]
